# Supplementary material for: Challenges Experienced and Observed during the Implementation of Leprosy Strategies, Sidama Region, Southern Ethiopia: An inductive thematic analysis of qualitative study among health professionals who working with leprosy programs
Source: PLoS Negl Trop Dis. 2023 Nov 30;17(11):e0011794. doi: 10.1371/journal.pntd.0011794 (PMC10715652; doi:10.1371/journal.pntd.0011794)
Supplement: Attachment — Submitted filename: Letter for reviwers.docx [file pntd.0011794.s001.docx]

**Kebede Tefera Betru (PhD, Assistant Professor)**

**Hawassa University School of Public Health**

**Ethiopia**

**PLOS Neglected Tropical Diseases.**

**14 September 2023**

**Subject: Response to Review Comments**

**Dear All Editors;**

**Greetings**

We hope this letter finds you well .We are delighted to submit the revised version of our manuscript titled “Challenges Experienced and Observed during the Implementation of Leprosy Strategies, Sidama Region, Southern Ethiopia: A qualitative Study” which was received positively by the reviewers. We would like to express our gratitude to the reviewers for providing insightful comments and suggestions that greatly enhanced the quality of our work. In this letter, we present a detailed response to each comment and describe the changes we have made in the manuscript accordingly.

Reviewer #1:

**1. Comment:** “Whave made some suggestions by line number here, but the author may want to have the paper reviewed by a native English speaker or work with the journal editor before publication for better clarity. There are a few minor content notes below also: Lines 44-49, 59-60, 60-61, 71-73, 108-109,114, 116-118, 119, 169,222,309 ,318,396 , 435 and 480"

**Response:** We greatly appreciate the reviewer's concern regarding the accessibility of our manuscript and have taken meticulous steps to address this issue. Specifically, we have made careful revisions to the language used throughout the manuscript to ensure it is easily understandable for a diverse readership. Additionally, we have substituted certain sections with recommendations to further enhance the manuscript's comprehensibility for a wider audience. We believe that these modifications will greatly contribute to making our manuscript more accessible to a broad range of readers..

Reviewer #2:

1. **Comment:** “Concerning the title, I suggest the authors include the approach thematic analysis and the general name for the participants as health professionals."

**Response:** We sincerely appreciate the reviewer's comment, and based on this suggestion, we have incorporated recommendations of research topic on the revised manuscript. ***Challenges Experienced and Observed during the Implementation of Leprosy Strategies, Sidama Region, Southern Ethiopia: An Inductive Thematic Analysis of Qualitative Study among Health Professionals who Working with Leprosy Programs***. This aid significantly enhances the understanding of our study area and improves the overall readability of the manuscript.

2. **Comment:** "At the introduction the authors should provide a brief background on the origin of leprosy. It is essential to mention Gerhard Armauer Hansen and his significant contribution to the discovery of the causal agent, Mycobacterium leprae."

**Response:** We sincerely appreciate the reviewer's observation. We have included a brief background on the origin of leprosy and Gerhard Armauer Hansen and his significant contribution to the discovery of the causal agent, Mycobacterium leprae. This addition provides readers with a solid foundation and context for our research .

3. **Comment:** “From lines 84 to 98, the authors should update the data related to leprosy using the most recent information available, such as the Weekly Epidemiological Record (WER), 9 September 2022, Vol. 97, No 36, 2022, pp. 429–452 [EN/FR]."

**Response:** We appreciate the reviewer's feedback .We agree with the reviewer's comment and have incorporated the suggested updated information.

**4. Comment:** “From lines 107 to 115, the authors should quote the total number of leprosy cases diagnosed in Ethiopia according to the latest reporting. Additionally, while mentioning the grade disability of 14%, they should clearly state that this percentage is underestimated and that expert-guided practice may reveal a higher value. They should also emphasize that MB cases represent more than 50% (1856) of new cases and are often associated with delayed diagnosis."

**Response**: We sincerely thank the reviewer for this valuable suggestion. In response, we have incorporated the total number of leprosy cases diagnosed in Ethiopia according to the latest reporting and indicated MB cases represent more than 50% of new cases and are often associated with delayed diagnosis by using reference (***de Lima AS, Pinto KC, Bona MPS, Mattos SML, Hoffmann MP, Mulinari-Brenner FA, Ottoboni VCD. Leprosy in a University Hospital in a Souther State of Brazil. An Bras Dermatol. 2015;90(5):654-9).*** This addition provides readers with a solid foundation and context for our research.

**5. Comment:** **“** A new subheading called "Type of Study and Site of Research" should be inserted. Under this heading, the authors should highlight the qualitative approach, specifically the "phenomenological approach." The period of data collection and site of research should be mentioned at the end of the sentence. “

**Response:** Thank you for pointing out this oversight. We have now inserted subheading called "Type of Study and Site of Research” and highlight given for the qualitative approach, specifically the "phenomenological approach." This adjustment should aid in making the manuscript more comprehensible to a broader audience.

**6. Comment:** “This section should follow a chronological order. Firstly, the authors should describe the sample size calculation used, which was based on data saturation.

Q: Are sufficient 12 interviewers for qualitative research? Maybe this reference will help you write a paragraph pointing out the answer for this question.

They can refer to the following source for more information: ***Hennink M, Kaiser BN. Sample sizes for saturation in qualitative research: A systematic review of empirical tests. Social science & medicine. 2022;292:114523.*** ***“***

**Response:** we appreciate the reviewer's comment, and we apologize for any confusion caused based on this. In the revised manuscript, we have included suggestions, including addressing the given reference.

**7. Comment:** “The authors should report on the purposive sampling method and the inclusion criteria for health workers at the selected and accessible health facilities who were sufficiently knowledgeable and experienced in leprosy case management and programs. They should also discuss how the participants were approached and the reasons for any refusals or dropouts. “

**Response:** we completely agree with your suggestion. We have carefully considered your comment and have revised the corresponding section of the manuscript accordingly.

**8. Comment:** “Lines 168 to 183 should be rewritten to include the ethical approval by the institutional committee and the approval number. The authors should explain why they chose to use verbal consent instead of written informed consent, and they should provide a rationale for this choice. “

**Response:** Thank you for your comment. But the raised comment was already addressed in lines 514-523. Moreover, we have taken into consideration and addressed some of the comments in the revised manuscript.

**9. Comment:** **“**The authors should also clarify who performed the interviews, why these individuals were chosen, and whether a pilot study was conducted to train the observers“

**Response:** we completely agree with your suggestion. We have carefully considered your comment and have revised the corresponding section of the manuscript accordingly.

**10. Comment:** “The instrument used for data collection (unstructured interview) should be described, along with details about the data collection site and any environmental interference during the interviews. Information about how the data was recorded and the application used should also be included, along with the duration of the interviews and how data saturation was determent. “

**Response**: we completely agree with your suggestion. We have carefully considered your comment and have revised the corresponding section of the manuscript accordingly.

**11. Comment:** “Regarding data analysis, line 185 should briefly explain why thematic analysis was chosen over other methods, such as grounded theory analysis. Additionally, the software used for analysis should be specified as [OpenCode 4.0 Umeå software (ICT Services and System Development and Division of Epidemiology and Global Health, Umea University, Sweden, 2015)] “

**Response:** Your suggestion has been fully acknowledged and we are in complete agreement with it. We have given detailed thought to your comment and as a result, we have made the necessary revisions to the relevant section of the manuscript.

**12 Comment:** Add a new column to report the number of health professionals with master's degrees in various fields (nurses, physicians, psychologists, etc.). Also, include a column reporting the amount of training on leprosy.

**Response**: We sincerely appreciate you pointing out these oversights. Your suggestion has been carefully considered, and we apologize for the uncollected data preventing us from making the required revisions to the corresponding section of the manuscript.

**13. Comment:** “Add a section on early diagnosis challenges in leprosy, which may involve sophisticated diagnostic tests. Mention that some cases with subclinical manifestations are diagnosed by molecular and immunological tests. Discuss the importance of a multidisciplinary team for early diagnosis. “

**Response:** Thank you for bringing this oversight to my attention. We have taken your suggestion into thorough consideration. We have made necessary revisions to the corresponding section of the manuscript.

**14. Comment:** “After line 416, remind the readers that leprosy diagnosis is also guided by laboratory exams such as baciloscopy, serology, and molecular analysis. Positive baciloscopy can confirm MB cases. “

**Response:** Thank you for bringing this oversight to my attention. we have taken your suggestion into thorough consideration. We have made necessary revisions to the corresponding section of the manuscript.

**15. Comment:** “From lines 417 to 423, provide the appropriate references. Consider discussing the differences between the ancestors of M. leprae and M. tuberculosis, their evolution, and how the diseases affect different body systems. “

**Response:** we completely agree with your suggestion. We have carefully considered your comment and have revised the corresponding section of the manuscript accordingly.

**16. Comment:** “In the conclusion section, clearly state that the challenges identified in the study can affect the implementation of leprosy prevention and control strategies. Re-evaluate the statement about achieving zero autochthonous cases by 2035, considering the persistent nature of M. leprae in societies. “

**Response:** We sincerely appreciate the reviewer's observation and we have taken your suggestion into thorough consideration. We have made necessary revisions to the corresponding section of the manuscript.

**17. Comment:** “The limitations of this research were not state“

**Response**: We sincerely appreciate the reviewer's comment, and based on this suggestion, we have incorporated the limitations of research on the revised manuscript.

In addition to addressing the specific comments raised by the reviewers, we have made numerous minor revisions throughout the manuscript to improve sentence structure, clarify certain passages, and correct typographical errors.

We sincerely hope that the revisions we have made address the reviewers' concerns and meet the standards of **PLOS Neglected Tropical Diseases**. We are confident that these modifications have strengthened our manuscript and made it more valuable to the scientific community. We would like to express our gratitude to the reviewers for their valuable feedback and the opportunity to improve our research.

Thank you for considering our revised submission. We remain available for any further clarifications or modifications as per your guidance. We appreciate your time and consideration.

**Sincerely,**

**Kebede Tefera Betru (PhD, Assistant Professor)**

[**ktefera2015@gmail.com**](mailto:ktefera2015@gmail.com)**;** [**kebedetefera@hu.edu.et**](mailto:kebedetefera@hu.edu.et) **or** [**64083055@mylife.unisa.ac.za**](mailto:64083055@mylife.unisa.ac.za)
