# Supplementary material for: Challenges Experienced and Observed during the Implementation of Leprosy Strategies, Sidama Region, Southern Ethiopia: An inductive thematic analysis of qualitative study among health professionals who working with leprosy programs
Source: PLoS Negl Trop Dis. 2023 Nov 30;17(11):e0011794. doi: 10.1371/journal.pntd.0011794 (PMC10715652; doi:10.1371/journal.pntd.0011794)
Supplement: Attachment — Submitted filename: Letter for reviwers.docx [file pntd.0011794.s002.docx]

**Kebede Tefera Betru (PhD, Assistant Professor)**

**Hawassa University School of Public Health**

**Ethiopia**

**PLOS Neglected Tropical Diseases.**

**10 November r 2023**

**Subject: Response to Review Comments**

**Dear Reviewer’s;**

**Greetings**

We hope this letter finds you well .We are delighted to submit the revised version of our manuscript titled "Challenges Experienced and Observed during the Implementation of Leprosy Strategies, Sidama Region, Southern Ethiopia: An Inductive Thematic Analysis of Qualitative Study among Health Professionals who Working with Leprosy Programs" which was received positively . We would like to thank the reviewer for offering informative remarks and ideas that significantly improved the quality of our work. In this letter, we respond to each remark and recommendation by describing the modifications we made to the manuscript accordingly.

**Reviewer #1:**

**1. Comment:** “In terms of the total number of participants, were this 23 or 23 plus an additional 12 HEWs. If I’m reading this correctly, 23 potential interviewees were selected by at 12, the authors reached data saturation? "

**Response:** We sincerely thanks and appreciate the reviewer's question, and we apologize for any confusion caused based on this.

The researchers selected a total of 23 participants (18 health workers and 5 health extension workers providing leprosy prevention and control program information and challenges. The interview continued until information saturation was reached. Therefore the study was reached saturation within twelve key informants of health workers and health extension workers in the study area.

**2. Comment**: Lines 50, 53, 80-81, 150, and 545-552,

Response: We appreciate the reviewer's feedback and in-depth observations .We agree with the reviewer's all lines comments and suggestions that have incorporated the suggested updated information and corrections. These adjustments should support in making the manuscript more comprehensible to a broader audience and improve the overall readability of the manuscript.

We sincerely hope that the revisions we have made address the reviewer's concerns and meet the standards of **PLOS Neglected Tropical Diseases**. We are confident that these modifications have strengthened our manuscript and made it more valuable to the scientific community. We would like to express our gratitude to the reviewer for their valuable feedback and the opportunity to improve our research.

Thank you for considering our revised submission. We remain available for any further clarifications or modifications as per your guidance. We appreciate your time and consideration.

**Sincerely,**

**Kebede Tefera Betru (PhD, Assistant Professor)**

[**ktefera2015@gmail.com**](mailto:ktefera2015@gmail.com)**;** [**kebedetefera@hu.edu.et**](mailto:kebedetefera@hu.edu.et) **or** [**64083055@mylife.unisa.ac.za**](mailto:64083055@mylife.unisa.ac.za)
